# 

**Published:** 1853-04-01

**Authors:** 


### DEATH OF DR. E. P. CHARLESWORTH.

(*From the Lancet.*)

"Dr. Charlesworth, who died at Lincoln, on February 20th, aged seventy-one, was son of the Rev. John Charlesworth, A.M., Fellow of Trinity College, Cambridge, and Rector of Ossington, Nottinghamshire. His grandfather was of the medical profession, of a family long resident in Nottinghamshire, formerly of Charlesworth, Derbyshire. The Doctor's medical education was begun under the pupilage of the late E. Harrison, M.D., of Horncastle, afterwards of Holles-street, London, and completed in Edinburgh, where he graduated in 1807. Having previously married, he at once settled in Lincoln. From Edinburgh he brought rare qualities. Gifted with an excellent memory, he was a close observer and a sound logician. No man could excel him in his clear, analytic power of reasoning; every thought was directed to some practical end. Though not a closet-student, he read much. He profoundly studied the book of nature; and no one had more deeply read mankind. With such qualities for forming a first-rate physician, no wonder he rose rapidly into repute, and acquired a wide practice in the county. He was early appointed physician to the County Hospital and Dispensary, besides giving gratuitous advice at his own house. To meet those increased demands upon his exertions, he became a perfect economist of time. Throughout life his early habits and the scrupulous exactness with which he fulfilled both public and private engagements, became proverbial. In consultation he was clear, careful, correct; his treatment of disease bold, but prudent; he never subjected his patient to rash experiment, nor pestered the medical attendant with multiplied remedies. His opinion was generally expressed in few words, for he had the power to speak, as he wrote, in aphorisms; and seldom was there room for dissent from his dictum; yet his deference and courtesy in canvassing an opposing opinion were remarkable; he delighted to discern merit in others; and one great aim of his life was to exalt, not to depreciate, his fellow-practitioners.

But we should be doing great injustice to the memory of Dr. Charlesworth, did we regard him merely in the capacity of an eminent and successful medical practitioner. Convinced that the well-being of public institutions depended upon strict supervision, and scrupulous performance of their duties by both officers and attendants, he was deemed by some a stern disciplinarian; but be it borne in mind, that he fought on the side of mercy and charity. He was a thorough reformer, and (like all reformers) was for awhile looked upon as a wild innovator, for to battle against ignorance and prejudice is to excite opposition.

The great work, however, of thirty of the best years of his life—"the labour of love" he laid out for himself—was his persistent effort to alleviate and improve the condition of those who suffer under the most dire and grievous affliction with which it pleases God to visit his creatures.

No sooner was the Lincoln Lunatic Asylum opened, than he was appointed one of the physicians. Impressed with the cruelty and mischief of the "brutal means of restraint" then practised at the Lincoln asylums, he conceived the grand idea to substitute moral control and kindness in the place of physical control and coercion! For years he had to contend against adverse boards, opposing colleagues, refractory officers, but his was the cause of refractory patients. Cautiously but vigorously—year by year—step by step—he proceeded. Leg-locks, and other instruments of torture, were hung up as obsolete curiosities in his library; strait-waistcoats were forbidden; restraint-chairs broken up; vigilant supervision by night and day was provided for; public inspection courted; every impediment in the way of coercion multiplied, until the imposition of restraint was more irksome to the attendant than to the patient. As his plan became developed, and his requirements from time to time obtained, Boards of Managements became more manageable and approving, and Hanwell, and other similar institutions, caught the enthusiasm, and the last relic of instrumental restraint vanished.

If France may justly boast giving birth to the man who had the humanity and courage first to strike the fetters from the raging maniac, England has no less right to be proud of him who had the wisdom and prescience to propound the maxim—"and out of love he taught it"—that moral restraint, gentleness, with firmness, were not only quite compatible with the *safety*, but were, indeed, the *true principles* on which the treatment of the insane should be conducted.

In social life, Dr. Charlesworth was most hospitable and courteous; his varied knowledge of men and things, his agreeable manners, and his animated and instructive conversation, made him a most fascinating companion."

---

### SUGGESTIONS WITH REFERENCE TO CASES OF INCURABLE LUNACY.

### To Correspondents.

THE following are among some of the valuable books, pamphlets, and reports received, and which want of space alone prevents us from noticing in this number. Mr. Haynes Walton on Ophthalmic Surgery; Dr. Cotton on Consumption; Dr. Fuller on Rheumatism, Gout, and Sciatica; Mr. Bishop on Deformities of the Human Body; Dr. Burgess on the Climate of Italy; Dr. Cockle on the Poison of the Cobra di Capello; Mr. F. W. Headland on the Action of Medicine; Mr. J. Wiblen and Dr. Harvey on the Yellow Fever; Dr. Knox on Art and Artistes; Morel's Traité Théorique et Pratique des Maladies Mentales; Reports of the State Lunatic Asylum, New York; and several Reports of Continental and English Asylums, &c., &c.; all of which will be reviewed in our July Number.

